# Supplementary material for: Cryo-EM Structure of the relaxosome, a complex essential for bacterial mating and the spread of antibiotic resistance genes
Source: Nat Commun. 2025 May 27;16:4906. doi: 10.1038/s41467-025-60116-6 (PMC12117103; doi:10.1038/s41467-025-60116-6)
Supplement: Supplementary file 1 — Supplementary Information [file 41467_2025_60116_MOESM1_ESM.pdf]

Supplementary Information

Cryo-EM Structure of the relaxosome, a complex essential for bacterial mating and the spread of antibiotic resistance genes

Sunanda M. Williams, Sandra Raffl, Sabine Kienesberger, Aravindan Ilangovan, Ellen L. Zechner, and Gabriel Waksman

Supplementary Table 1a. Cryo-EM data collection refinement and validations statistics and supplementary information. The supplementary information part of this table provides information on how each map was used, the part(s) that were built in each map when relevant, the method that was used to build the model from each map when relevant, and the PDB entry code of the corresponding model.

**Complex/ Subcomplex**					**Relaxosome**					
Name of the maps generated in this study and Resolution	ds$_{-27_+143}$-R Locally-refined Map 3.78 Å	ds$_{-27_+143}$-R Global Map 4.31 Å	ss$_{-27_+8}$ds$_{+9_+143}$-R Locally-refined Map 3.45 Å	ss$_{-27_+8}$ds$_{+9_+143}$-R Global Map 3.77 Å	ss$_{-27_+8}$ds$_{+9_+143}$ -RΔ$_{TraM}$ Locally-refined Map 2.94 Å	ss$_{-27_+8}$ds$_{+9_+143}$ -RΔ$_{TraM}$ Global Map 3.11 Å	ss$_{-27_+8}$ds$_{+9_+143}$ -RΔ$_{AH+CTD}$ Locally-refined Map 3.42Å	ss-$_{27_+8}$ds$_{+9_+143}$ -RΔ$_{AH+CTD}$ Global Map 3.93 Å	ss$_{-27_+3}$ ds$_{+4_+143}$-R Locally-refined Map 3.68 Å	ss$_{-27_-3}$ ds$_{-2_+143}$-R Locally-refined Map 3.42 Å
EMDB entry	EMD-50117	EMD-50131	EMD-50118	EMD-53548	EMD-50120	EMD-50133	EMD-50119	EMD-50132	EMD-50121	EMD-50122
Data collection facility	ISMB and eBIC	ISMB and eBIC	ISMB	ISMB	eBIC	eBIC	eBIC	eBIC	ISMB	ISMB
**Data collection and processing**										
Magnification	81,000	81,000	105,000	105,000	105,000	105,000	105,000	105,000	105,000	105,000
Voltage (kV)	300	300	300	300	300	300	300	300	300	300
Total exposure (e^-/Å^2)	50	50	50	50	50	50	50	50	50	47
Defocus range (µm)	1.5 to -3.3	1.5 to -3.3	-1.5 to -2.7	-1.5 to -2.7	-0.9 to -2.7	-0.9 to -2.7	-0.9 to -2.7	-0.9 to -2.7	0.9 to -2.4	-1.2 to -2.4
Pixel size (Å)	1.06	1.06	0.828	0.828	0.825	0.825	0.825	0.825	0.828	0.828
Symmetry imposed	C1	C1	C1	C1	C1	C1	C1	C1	C1	C1

No. of Movies	55,914	55,914	11,276	11,276	30,675	30,675	29,203	29,203	21,148	16,404
Initial particle images (no.)	20,752,847	20,752,847	3,830,425	3,830,425	7,940,692	7,940,692	8,381,227	8,381,227	7,055,608	7,065,597
Final particle images (no.)	337,238	337,238	155,077	155,077	330,708	330,708	165,577	165,577	159,080	177,743
Map resolution (Å)	3.78 Å	4.31 Å	3.45 Å	3.77 Å	2.94 Å	3.11 Å	3.42Å	3.93 Å	3.68 Å	3.42 Å
FSC threshold	0.143	0.143	0.143	0.143	0.143	0.143	0.143	0.143	0.143	0.143
**Additional information**										
Map was used to	Build the structure of ds_{-27_+143}-R	As an input map and mask creation for local refinement, to generate the locally-refined ds_{-27_+143}-Rmap	Build the structure of $ss_{-27\_+8}ds_{+9\_+143}$-R	1. As an input map and mask creation for local refinement, to generate the locally-refined $ss_{-27\_+8}ds_{+9\_+143}$-R map. 2. Fit NTD and 1A sub-domains of VH, all the sub-domains of AH, CTD and TraM	Build the structure of $ss_{-27\_+8}ds_{+9\_+143}$-R _ΔTraM	1. As an input map and mask creation for local refinement, to generate the locally-refined $ss_{-27\_+8}ds_{+9\_+143}$-R _ΔTraM map. 2. To confirm the location of TraM in the relaxosome structure.	Build the structure of $ss_{-27\_+8}ds_{+9\_+143}$-R _ ΔAH+CTD	1. As an input map and mask creation for local refinement, to generate the locally-refined $ss_{-27\_+8}ds_{+9\_+143}$-R _ ΔAH+CTD map. 2. To confirm the region of TraI AH+CTD in the relaxosome structure.	Build the structure of $ss_{-27\_+3}ds_{+4\_+143}$-R	Build the structure of $ss_{-27\_-3}ds_{-2\_+143}$-R
Part(s) built in the map	R-and T-strand DNA (+12 to +94), IHF αβ, TraYs 1,2 and 3, TraI	N/A	R-strand (+10 to +93) and T-strand DNA (-2 to +93), IHF αβ, TraYs 1,2 and 3,	The entire $ss_{-27\_+8}ds_{+9\_+143}$-R structure, VH_{NTD}, VH_{1A}, complete AH domain, TraM/DNA	R-strand (+10 to +94) and T-strand DNA (-2 to +94), IHF αβ, TraYs 1,2 and 3,	N/A	R-strand (+10 to +94) and T-strand DNA (-2 to +94), IHF αβ, TraYs 1,2 and 3, TraI TE	N/A	R-strand (+10 to +94) and T-strand DNA (-2 to +94), IHF αβ, TraYs 1,2	R-strand (+10 to +94) and T-strand DNA (-2 to +94), IHF αβ, TraYs 1,2 and 3, TraI TE

	$VH_{2A+2B/2B\text{-}like}$ sub-domain		TraI TE and $VH_{2A+2B/2B\text{-}like}$		TraI TE and $VH_{2A+2B/2B\text{-}like}$		and $VH_{2A+2B/2B\text{-}like}$		and 3, TraI TE and $VH_{2A+2B/2B\text{-}like}$	and $VH_{2A+2B/2B\text{-}like}$
Modelling method	Building and refinement	N/A	Building and refinement	Rigid body	Building and refinement	N/A	Building and refinement	N/A	Building and refinement	Building and refinement
PDB deposited	9F0X	N/A	9F0Y	Coordinates are included in a ChimeraX session as supplementary information	9F10	N/A	9F0Z	N/A	9F11	9F12

Supplementary Table 1b. Model building and validation statistics

**Complex/Subcomplex**	**Relaxosome**					
Structures generated in this study	ds_{-27_+143}-R	$ss_{-27\_+8}ds_{+9\_+143}$-R	$ss_{-27\_+8}ds_{+9\_+143}$-RΔ_{TraM}	$ss_{-27\_+8}ds_{+9\_+143}$-RΔ_{AH+CTD}	$ss_{-27\_+3}ds_{+4\_+143}$-R	$ss_{-27\_-3}ds_{-2\_+143}$-R
PDB entry	9F0X	9F0Y	9F10	9F0Z	9F11	9F12
**Refinement**						
EMDB corresponding	50117	50118	50120	50119	50121	50122
Initial model used (PDB code)	*de novo*	ds_{-27_+143}-R (PDB ID: 9F0X)	$ss_{-27\_+8}ds_{+9\_+143}$-R (PDB ID: 9F0Y)	$ss_{-27\_+8}ds_{+9\_+143}$-R (PDB ID: 9F0Y)	$ss_{-27\_+8}ds_{+9\_+143}$-R (PDB ID: 9F0Y)	$ss_{-27\_+8}ds_{+9\_+143}$-R (PDB ID: 9F0Y)
Model resolution (Å)	3.87	3.13	3.03	3.18	3.66	3.16
FSC threshold	0.5	0.5	0.5	0.5	0.5	0.5
CC Model vs. Data (mask)	0.71	0.85	0.82	0.74	0.73	0.85
**Model composition**						
Nonhydrogen atoms	9,423	11,968	12,286	11,990	11,950	12,172
Protein residues	791	1,083	1,094	1,074	1,067	1,091
Nucleotide residues	166	179	181	181	181	181
Ligands	-	1	1	1	1	1
**B factors (Å^2)**						
Protein	89.0 (49.5-163.6)	77.4 (30.0-195)	63.1 (25.7-155.3)	81.12 (21.9-164.6)	88.6 (30.0-135.6)	86.38 (30.0-166.6)
Nucleotide	107.91 (65.0-217.3)	82.15 (20-224.4)	57.2 (20.0-181.7)	83.0 (20.0-264.5)	92.8 (20.0-230.9)	91.7 (20.0-235.5)
Ligand	-	46.48	34.25	94.0	99.15	53.29
**R.m.s. deviations**						
Bond lengths (Å)	0.005	0.006	0.005	0.005	0.005	0.004
Bond angles (°)	0.636	0.662	0.645	0.655	0.681	0.599
**Validation**						
MolProbity score	1.54	1.48	1.42	1.42	1.34	1.20
Clashscore	5.42	5.44	5.34	5.37	3.94	2.78
Rotamer outliers (%)	0.81	0	0.11	0.58	0.23	0.23
**Ramachandran plot**						
Favored (%)	96.25	96.88	97.30	97.29	97.11	97.30
Allowed (%)	3.75	3.12	2.7	2.71	2.89	2.7
Disallowed (%)	0	0	0	0	0	0

Supplementary Table 2. Cryo-EM data collection statistics for datasets used to generate 3D maps to illustrate DNA melting by the relaxosome.

**Complex/Subcomplex**	**Relaxosome**					
Name of complex	ds$_{-27_+143}$-R	ss$_{-27_+8}$ds$_{+9_+143}$-R	ss$_{-27_-8}$ds$_{-7_+143}$-R	ss$_{-27_-13}$ds$_{-12_+143}$-R	ds$_{-2_+113}$-R	ds$_{-67_+113}$(poly-dT15 $_{-17\_-3}$)-R
Data collection facility			ISMB	ISMB	ISMB	ISMB
**Data collection and processing**						
Magnification			105,000	105,000	105,000	105,000
Voltage (kV)			300	300	300	300
Total exposure ($e^-/Å^2$)	See Table S1A		48.9	50	50.8	49.3
Defocus range (µm)			-1.2 to -2.4	-1.2 to -2.7	-0.9 to -2.4	0.9 to -2.4
Pixel size/(Å)			0.828	0.828	0.828	0.828
No. of Movies			14,575	18,697	10,778	13,229
Initial particle images (no.)			6,565,218	5,432,852	3,280,832	4,251,001
Final particle images (no.)	212,673	369,134	31,342	338,844	202,962	346,208
Resolution	6.34	3.81 low pass filtered to 10	7.70	6.50	6.14	4.43 low pass filtered to 7
EMDB entry	EMD-50098	EMD-50099	EMD-50102	EMD-50103	EMD-50104	EMD-50105

Supplementary Table 3. Functional analysis of *traI* mutants. Donor cells carrying the plasmids in the first column were mated with *E. coli* MS614 for 60 minutes. Average number of transconjugants obtained relative to donor cells is shown for three independent experiments. Standard deviations for the mean values are indicated. P-values were determined with ordinary one way anova, Holm-Šídák's multiple comparisons test comparing wild-type and mutants frequencies within respective groups. P-values <0.05 were considered significant. Source data are provided as a Source Data file.

		**[a]Conjugation frequency**		
	**Variant of F TraI**	**Mean**	**SD**	**P-value**
**Donor *E. coli* MS411 [pOX38]**				
pTrc99A	vector	2.93×10^{-1}	0.1520	n.a.
**Donor *E. coli* MS411 [pOX38Δ*traI*]**				
[b]p99I$^+$	wild-type	1.11×10^{-3}	0.0007	---
**Donor *E. coli* MS411 [pOX38Δ*traI*]**				
	**DNA binding**			
p99I$^+$_Mut3	R576A R605A	5.89×10^{-4}	0.0003	>0.9999
p99I$^+$_Mut4	R576E R605E	2.97×10^{-3}	0.0014	>0.9999
p99I$^+$_Mut5	S641E S696E	8.45×10^{-1}	0.3002	<0.0001
p99I$^+$_Mut6	R694E S696E	6.53×10^{-1}	0.1659	0.0001
p99I$^+$_Mut7	S641E R694E S696E	6.13×10^{-1}	0.1834	0.0002
p99I$^+$_Mut11	R651E N799E	8.20×10^{-4}	0.0005	>0.9999
	**TraI-TraY**			
p99I$^+$_Mut12	L646E R647E	2.13×10^{-3}	0.0008	0.8778
p99I$^+$_Mut13	L646R D648R	3.74×10^{-2}	0.0158	0.0016
	**TraI-IHF**			
p99I$^+$_Mut14	S678K S680R	1.56×10^{0}	0.3140	<0.0001
p99I$^+$_Mut15	R673E T675E	5.23×10^{-2}	0.0217	0.6636
p99I$^+$_Mut16	R673E T675E Q677E	2.01×10^{-1}	0.0106	0.2139
	**TE-VH**			
p99I$^+$_Mut17	S73E W659E	1.24×10^{0}	0.6521	0.0181
p99I$^+$_Mut18	S700E S701E	9.51×10^{-1}	0.5485	0.0293

[a]Transconjugants/donor n=3; [b]n=4

Supplementary Table 4. Strains, plasmids, constructs and oligonucleotides used in this study.

a. Strains used in this study

E. coli Strains	Description	Reference/Source
Top10	F- mcrA Δ(mrr-hsdRMS-mcrBC) Φ80lacZΔM15 Δ lacX74 recA1 araD139 Δ(araleu)7697 galU galK rpsL (StrR) endA1 nupG. Used for cloning.	Invitrogen
BL21(DE3)	F^- ompT hsdSB (rB^-, mB^-) galdcmrne131 (DE3) Used for overexpression of proteins.	Invitrogen
C41(DE3)	BL21 (DE3 *[lacI lac-T7 gene 1 ind1 sam7 nin5]*) Used for overexpression of toxic proteins.	Lucigen
MS411	*ilvG rfb-50 thi*	M. Schembri; DTU, Denmark
MS614	Sm^R; *ilvG, rfb-50, thi, rpsL*	M. Schembri; DTU, Denmark
DH5α	*endA1 recA1 gyrA96 thi-l hsdR17 supE44 λ- relA1 deoR Δ(lacZYAargF)-U169 φ80dlacZΔ*(M15)	[1]

b. Plasmids used in this study

Plasmids	Description	Reference/Source
pRSF-1b	$P_{T7\ RNAP}$ Amp^R expression vector with RSF1030 replication origin	Novagen
pET28a(+)	$P_{T7\ RNAP}$ Km^R expression vector with pBR322 origin	Novagen
pUC57	High copy number cloning vector P_{lac} Amp^R pMB1 origin	Genscript
pUC57-mini	High copy number cloning vector Amp^R minimal pUC57 without LacZ	Genscript
pCR-BluntII-TOPO vector	lacZα-ccdB Km^R Zeo^R pUC origin *Vaccinia virus* TopoisomeraseI covalently attached to 3' ends	ThermoFisher
pOX38	Km^R; IncFI, derivative of F r;	[2]
pOX38ΔtraI	Km^R, Tc^R; IncFI, *traI::tetRA*	[3]
pTrc99A	Amp^R; cloning vector; *trc* promoter; *lacI*^{q}	[4]
p99I$^+$	Amp^R; pTrc99A with wild-type F *traI*	[5]

c. Constructs used in this study

Plasmid	Description/Source	Primer Name*	Source/Reference
pRSF-IHF	IHF cloned and expressed without tags in pRSF-1b plasmid	Nt-IHF_F<br>Nt-IHF_R	This study
pET28a-TraY	TraY cloned and expressed without tags in pET28a plasmid	-	Genscript
pET28a-TraM	TraM cloned and expressed without tags in pET28a plasmid	-	Genscript
pTrc99A -TraI	F plasmid TraI cloned and expressed without tag in pTrc99A plasmid	-	[5]

pTrc99A-Y16FTraI	Changes codon 16 of F plasmid TraI from TAT (Y) to TTT (F) to yield F TraI mutant Y16F	pTrc99a_TraI_Y16F_F<br>pTrc99a_TraI_Y16F_R	This study
pTrc99A-Y16FTraI$^{1-863}$	Truncated Y16FTraI containing 1-863 amino acids	Y16FtraI_delAH+CTD_F<br>Y16FtraI_delAH+CTD_R	This study
pUC57-oriT316	Large-scale PCR amplification of *dsoriT316*	ForiT_for<br>ForiT_rev	This study
pUC57-mini-oriT170_5X	Vector with five repeats of 170 bp oriT. Used for large-scale restriction digestion to generate ds$_{-27_+143}$	-	Genscript
p99I$^+$_Mut3	p99I$^+$ with F *traI* mutant R576A R605A	MutI fw<br>MutI rev<br>R576A fw<br>R576A rev<br>R605A fw<br>R605A rev	This study
p99I$^+$_Mut4	p99I$^+$ with F *traI* mutant R576E R605E	MutI fw<br>MutI rev<br>R576E fw<br>R576E rev<br>R605E fw<br>R605E rev	This study
p99I$^+$_Mut5	p99I$^+$ with F *traI* mutant S641E S696E	MutI fw<br>MutI rev<br>S641E fw<br>S641E rev<br>S696E fw<br>S696Erev	This study
p99I$^+$_Mut6	p99I$^+$ with F *traI* mutant R694E S696E	MutI fw<br>MutI rev<br>R694E S696E fw<br>R694E S696E rev	This study
p99I$^+$_Mut7	p99I$^+$ with F *traI* mutant S641E S696E R694E	MutI fw<br>MutI rev<br>S641E fw<br>S641E rev<br>R694E S696E fw<br>R694E S696E rev	This study
p99I$^+$_Mut11	p99$^+$ with F *traI* mutant R651E N799E	MutI fw<br>MutI rev<br>R651E fw<br>R651E rev<br>N799E fw<br>N799Erev	This study
p99I$^+$_Mut12	p99$^+$ with F *traI* mutant L646E R647E	MutI fw<br>MutI rev<br>L646E R647E fw<br>L646E R647E rev	This study
p99I$^+$_Mut13	p99$^+$ with F *traI* mutant L646R D648R	MutI fw<br>MutI rev<br>L646R D648R fw<br>L646R D648R rev	This study
p99I$^+$_Mut14	p99$^+$ with F *traI* mutant S678K S680R	MutI fw<br>MutI rev<br>S678K S680R fw<br>S678K S680R rev	This study

p99I⁺_Mut15	p99⁺ with F *traI* mutant R673E T675E	MutI fw<br>MutI rev<br>R673E T675E fw<br>R673E T675E rev	This study
p99I⁺_Mut16	p99⁺ with F *traI* mutant R673E T675E Q677E	MutI fw<br>MutI rev<br>R673E T675E Q677E fw<br>R673E T675E Q677E rev	This study
p99I⁺_Mut17	p99⁺ with F *traI* mutant S73E W659E	MutI17fw<br>MutI rev<br>S73E fw<br>S73E rev<br>W659E fw<br>W659E rev	This study
p99I⁺_Mut18	p99⁺ with F *traI* mutant S700E S701E	MutI fw<br>MutI rev<br>S700E S701E fw<br>S700E S701E rev	This study

* '_F', '_for' or 'fw' suffix for primer names denote the forward primer and '_R' or '_rev' denotes the reverse primer

d. Sequences of primers used in this study

**Primer name**	**Primer Sequence (5'-3')** *
Nt-IHF_F	tcagcttttgtaagcgccatggtatatc
Nt-IHF_R	tttaactttaataaggagatataccatggcgc
pTrc99a_TraI_Y16F_F	cgggaacttttataccgacaaggataattac
pTrc99a_TraI_Y16F_R	gtataaaagttcccggcacttccgg
Y16FtraI_delAH+CTD_F	acacgccgtgatctagagtcgacctgcagg
Y16FtraI_delAH+CTD_R	tagatcacggcgtgtgaagtgcggac
ForiT_for	ataatgcaaacagggacgcaccgct
ForiT_rev	ctccacaaaaaggctcaacaggttg
MutI fw	ccgcacgtcataacgtacaggtcctgataaccgacagc
MutI rev	gaccgcacggtgtgtgggacccagccccacgactcggg
MutI17fw	gaaacagaccatggaattcgagctc
R576A fw	cagtgaaccggacGCtaatgtcc
R576A rev	ggacattaGCgtccggttcactg
R605A fw	cagcggggtaGCggaacagg
R605A rev	cctgttccGCtaccccgctg
R576E fw	cagtgaaccggacGAGaatgtcc
R576E rev	ggacattCTCgtccggttcactg
R605E fw	cagcggggtaGAggaacagg
R605E rev	cctgttccTCtaccccgctg
S641E fw	ctggacGAGcggagccgttatc
S641E rev	gataacggctccgCTCgtccag
S696E fw	gtggtgcgtattGAGtccctgg
S696E rev	ccagggaCTCaatacgcaccac
R694E S696E fw	gtggtgGAGattGAGtccctggacagcagc
R694E S696E rev	gctgctgtccagggaCTCaatCTCcaccac
S641E fw	ctggacGAGcggagccgttatc
S641E rev	gataacggctccgCTCgtccag
R651E fw	ggatatgtacGAGccggggatgg
R651E rev	ccatccccggCTCgtacatatcc
N799E fw	caatggacGaGgccaccctgaac
N799Erev	gttcagggtggcCtCgtccattg

L646E R647E fw	agccgttatGAgGAggatatgtaccg
L646E R647E rev	cggtacatatccTCcTCataacggct
L646R D648R fw	ccgttatcGgcggCGtatgtacc
L646R D648R rev	ggtacataCGccgcCgataacgg
S678K S680R fw	ggcgcagaAAcacagActgaccctg
S678K S680R rev	cagggtcagTctgtgTTtctgcgcc
R673E T675E fw	gttatcgacGAggtgGAggcgcagagtc
R673E T675E rev	gactctgcgccTCcaccTCgtcgataac
R673E T675E Q677E fw	gttatcgacGAggtgGAggcgGagagtcacag
R673E T675E Q677E rev	ctgtgactctCcgccTCcaccTCgtcgataac
S73E fw	catgcaggatggcGAGaacaggc
S73E rev	gcctgttCTCgccatcctgcatg
W659E fw	gatggagcagGAgaacccggagac
W659E rev	gtctccgggttcTCctgctccatc
S700E S701E fw	ccctggacGAGGAGtggtcgctgttc
S700E S701E rev	gaacagcgaccaCTCCTCgtccaggg

* Mutated bases are shown in capital letters

e. *oriT* DNA sequences:

**oriT DNA**		**Sequence (5' – 3')**
dsoriT316	R- strand	ctccacaaaaaggctcaacaggttggtggttctcaccaccaaaagcaccacaccccacgcaaaaacaagtttt<br>tgctgattttctttataaatagagtgttatgaaaaattagtttctcttactctctttatgatatttaaaaaagcggtg<br>tcggcgcggctacaacaacgcgccgacaccgttttgtaggggtggtactgactattttataaaaaacattattt<br>atattaggggtgctgctagcggcgcggtgtgtttttttataggataccgctaggggcgctgctagcggtgcgtcc<br>ctgtttgcattat
	T- strand	ataatgcaaacagggacgcaccgctagcagcgcccctagcggtatcctataaaaaaacacaccgcgccgcta<br>gcagcacccctaatataaaataatgtttttataaaaatagtcagtaccacccctacaaaacggtg<br>tcggcgcgttgttgtagccgcgccgacaccgctttttaaatatcataaagagagtaagagaaactaattttcat<br>aacactctatttataaagaaaaatcagcaaaaacttgtttttgcgtggggtgtggtgcttttggtggtg<br>agaaccaccaacctgttgagcctttttgtggag
ds$_{-27\_+143}$	R- strand	ttggtggttctcaccaccaaaagcaccacaccccacgcaaaaacaagtttttgctgattttctttataaatagag<br>tgttatgaaaaattagtttctcttactctctttatgatatttaaaaaagcggtgtcggcgcggctacaac<br>aacgcgccgacaccgttttgtagg
	T- strand	cctacaaaacggtgtcggcgcgttgttgtagccgcgccgacaccgctttttaaatatcataaagagagtaaga<br>gaaactaattttcataacactctatttataaagaaaaatcagcaaaaacttgtttttgcgtggggtgtg<br>gtgcttttggtggtgagaaccaccaa
ss$_{-27\_+8}$*ds*$_{+9\_+143}$	R- strand	cgcaaaaacaagtttttgctgattttctttataaatagagtgttatgaaaaattagtttctcttactctctttatgat<br>atttaaaaaagcggtgtcggcgcggctacaacaacgcgccgacaccgttttgtagg
	T- strand	cctacaaaacggtgtcggcgcgttgttgtagccgcgccgacaccgctttttaaatatcataaagagagtaaga<br>gaaactaattttcataacactctatttataaagaaaaatcagcaaaaacttgtttttgcgtggggtgtggt<br>gcttttggtggtgagaaccaccaa
ss$_{-27\_+3}$*ds*$_{+4\_+143}$	R- strand	ccccacgcaaaaacaagtttttgctgattttctttataaatagagtgttatgaaaaattagtttctcttactctcttt<br>atgatatttaaaaaagcggtgtcggcgcggctacaacaacgcgccgacaccgttttgtagg
	T- strand	cctacaaaacggtgtcggcgcgttgttgtagccgcgccgacaccgctttttaaatatcataaagagagtaaga<br>gaaactaattttcataacactctatttataaagaaaaatcagcaaaaacttgtttttgcgtggggtgtgg<br>tgcttttggtggtgagaaccaccaa
ss$_{-27\_-3}$*ds*$_{-2\_+143}$	R- strand	ccacaccccacgcaaaaacaagtttttgctgattttctttataaatagagtgttatgaaaaattagtttctcttac<br>tctctttatgatatttaaaaaagcggtgtcggcgcggctacaacaacgcgccgacaccgttttgtagg
	T- strand	cctacaaaacggtgtcggcgcgttgttgtagccgcgccgacaccgctttttaaatatcataaagagagtaaga<br>gaaactaattttcataacactctatttataaagaaaaatcagcaaaaacttgtttttgcgtggggtgtgg<br>tgcttttggtggtgagaaccaccaa
ss$_{-27\_-8}$*ds*$_{-7\_+143}$	R- strand	aagcaccacaccccacgcaaaaacaagtttttgctgattttctttataaatagagtgttatgaaaaattagtttc<br>tcttactctctttatgatatttaaaaaagcggtgtcggcgcggctacaacaacgcgccgacaccgttttgt<br>agg

	T- strand	cctacaaaacggtgtcggcgcgttgttgtagccgcgccgacaccgctttttaaatatcataaagagagtaagagaaactaattttcataacactctatttataaagaaaaatcagcaaaaacttgtttttgcgtggggtgtggtgcttttggtggtgagaaccaccaa
ss$_{-27_-13}$ds$_{-12_+143}$	R- strand	accaaaagcaccacaccccacgcaaaaacaagtttttgctgatttttctttataaatagagtgttatgaaaaattagtttctcttactctctttatgatatttaaaaaagcggtgtcggcgcggctacaacaacgcgccgacaccgttttgtagg
	T- strand	cctacaaaacggtgtcggcgcgttgttgtagccgcgccgacaccgctttttaaatatcataaagagagtaagagaaactaattttcataacactctatttataaagaaaaatcagcaaaaacttgtttttgcgtggggtgtggtgcttttggtggtgagaaccaccaa
ds$_{-2_+113}$	R- strand	ccacaccccacgcaaaaacaagtttttgctgatttttctttataaatagagtgttatgaaaaattagtttctcttactctctttatgatatttaaaaaagcggtgtcggcgcggc
	T- strand	gccgcgccgacaccgctttttaaatatcataaagagagtaagagaaactaattttcataacactctatttataaagaaaaatcagcaaaaacttgtttttgcgtggggtgtgg
ds$_{-67_+113}$(poly-dT15$_{-17_-3}$)	R- strand	cgtaaataatttaacccactccacaaaaaggctcaacaggttggtggttc-ttttttttttttttt-ccacaccccacgcaaaaacaagtttttgctgatttttctttataaatagagtgttatgaaaaattagtttctcttactctctttatgatatttaaaaaagcggtgtcggcgcggc
	T- strand	gccgcgccgacaccgctttttaaatatcataaagagagtaagagaaactaattttcataacactctatttataaagaaaaatcagcaaaaacttgtttttgcgtggggtgtgg-tgcttttggtggtga-gaaccaccaacctgttgagcctttttgtggagtgggttaaattatttacg

Region in blue is unhybridized in the final construct

f. Relaxosome footprints sequence:

**Name**	**Footprint sequence (5'-3' on R strand)**
ft-57bp	TGACTATTTTTATAAAAAACATTATTTTATATTAGGGGTGCTGCTAGCGGCGCGGTG
ft-47bp	TGAAAAATTAGTTTCTCTTACTCTCTTTATGATATTTAAAAAAGCGG
ft-74bp	TGATTTTTCTTTATAAATAGAGTGTTATGAAAAATTAGTTTCTCTTACTCTCTTTATGATATTTAAAAAAGCGG
ft-93bp	CGCAAAAACAAGTTTTTGCTGATTTTTCTTTATAAATAGAGTGTTATGAAAAATTAGTTTCTCTTACTCTCTTTATGATATTTAAAAAAGCGG
ft-97bp	CACGCAAAAACAAGTTTTTGCTGATTTTTCTTTATAAATAGAGTGTTATGAAAAATTAGTTTCTCTTACTCTCTTTATGATATTTAAAAAAGCGGTG
ft-98bp	CACCCCACGCAAAAACAAGTTTTTGCTGATTTTTCTTTATAAATAGAGTGTTATGAAAAATTAGTTTCTCTTACTCTCTTTATGATATTTAAAAAAGC
ft-100bp	CACCCCACGCAAAAACAAGTTTTTGCTGATTTTTCTTTATAAATAGAGTGTTATGAAAAATTAGTTTCTCTTACTCTCTTTATGATATTTAAAAAAGCGG
ft-101bp	ACACCCCACGCAAAAACAAGTTTTTGCTGATTTTTCTTTATAAATAGAGTGTTATGAAAAATTAGTTTCTCTTACTCTCTTTATGATATTTAAAAAAGCGG
ft-103bp	CAAAAACAAGTTTTTGCTGATTTTTCTTTATAAATAGAGTGTTATGAAAAATTAGTTTCTCTTACTCTCTTTATGATATTTAAAAAAGCGGTGTCGGCGCGGC
ft-112bp	CACCCCACGCAAAAACAAGTTTTTGCTGATTTTTCTTTATAAATAGAGTGTTATGAAAAATTAGTTTCTCTTACTCTCTTTATGATATTTAAAAAAGCGGTGTCGGCGCGGC
ft-128bp	TCACCACCAAAAGCACCACACCCCACGCAAAAACAAGTTTTTGCTGATTTTTCTTTATAAATAGAGTGTTATGAAAAATTAGTTTCTCTTACTCTCTTTATGATATTTAAAAAAGCGGTGTCGGCGCG
ft-66bp	TGTTATGAAAAATTAGTTTCTCTTACTCTCTTTATGATATTTAAAAAAGCGGTGTCGGCGCGGCTA
ft-89bp	AAAACAAGTTTTTGCTGATTTTTCTTTATAAATAGAGTGTTATGAAAAATTAGTTTCTCTTACTCTCTTTATGATATTTAAAAAAGCGG
ft-94bp_1	CCACGCAAAAACAAGTTTTTGCTGATTTTTCTTTATAAATAGAGTGTTATGAAAAATTAGTTTCTCTTACTCTCTTTATGATATTTAAAAAAGC
ft-94bp_2 (X 2)	GCAAAAACAAGTTTTTGCTGATTTTTCTTTATAAATAGAGTGTTATGAAAAATTAGTTTCTCTTACTCTCTTTATGATATTTAAAAAAGCGGTG
ft-118bp	ACACCCCACGCAAAAACAAGTTTTTGCTGATTTTTCTTTATAAATAGAGTGTTATGAAAAATTAGTTTCTCTTACTCTCTTTATGATATTTAAAAAAGCGGTGTCGGCGCGGCTACAA

g. DNA and proteins used for relaxosome complex reconstitution

**Relaxosome complex**	***oriT* DNA**	**IHF and Accessory proteins**	**TraI variant**
ds_{-27_+143}-R	ds_{-27_+143}	IHF, TraM and TraY	Y16FTraI
$ss_{-27\_+8}ds_{+9\_+143}$-R	$ss_{-27\_+8}ds_{+9\_+143}$	IHF, TraM and TraY	Y16FTraI
$ss_{-27\_+8}ds_{+9\_+143}$-$R\Delta_{TraM}$	$ss_{-27\_+8}ds_{+9\_+143}$	IHF and TraY	Y16FTraI
$ss_{-27\_+8}ds_{+9\_+143}$-$R\Delta_{AH+CTD}$	$ss_{-27\_+8}ds_{+9\_+143}$	IHF, TraM and TraY	$Y16FTraI_{\Delta AH+CTD}$
$ss_{-27\_+3}ds_{+4\_+143}$-R	$ss_{-27\_+3}ds_{+4\_+143}$	IHF, TraM and TraY	Y16FTraI
$ss_{-27\_-3}ds_{-2\_+143}$-R	$ss_{-27\_-3}ds_{-2\_+143}$	IHF, TraM and TraY	Y16FTraI
$ss_{-27\_-8}ds_{-7\_+143}$-R	$ss_{-27\_-8}ds_{-7\_+143}$	IHF, TraM and TraY	Y16FTraI
$ss_{-27\_-13}ds_{-12\_+143}$-R	$ss_{-27\_-13}ds_{-12\_+143}$	IHF, TraM and TraY	Y16FTraI
ds_{-2_+113}-R	ds_{-2_+113}	IHF and TraY	Y16FTraI
ds_{-67_+113}(poly-dT15$_{-17_-3}$)-R	ds_{-67_+113}(poly-dT15$_{-17_-3}$)	IHF and TraY	Y16FTraI

Supplementary Figures

Supplementary Fig. 1

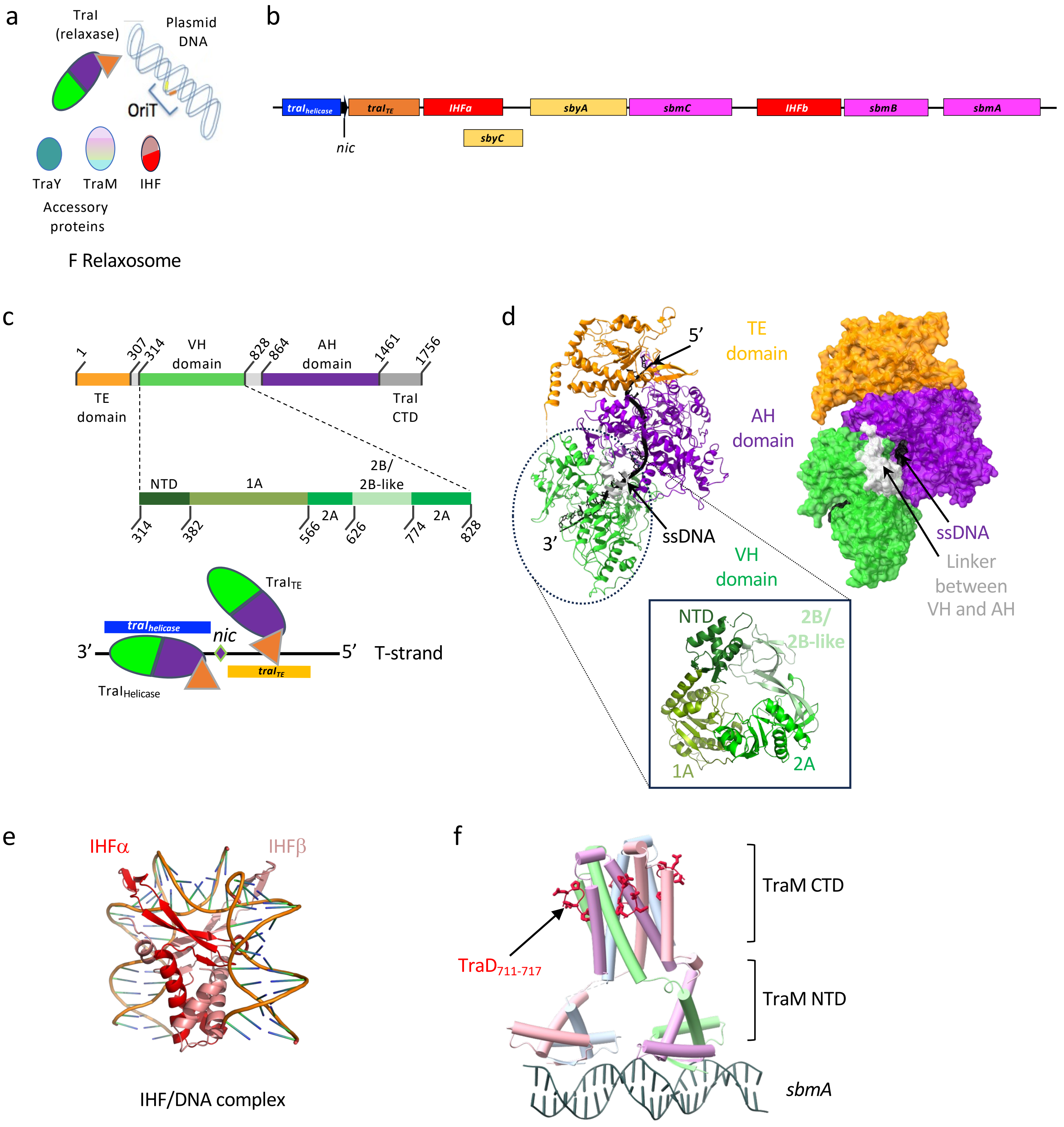

Supplementary Fig. 1. Prior knowledge on the structure of relaxosome components.

a, F plasmid relaxosome components.

b, *oriT* region of the F plasmid. The binding sites for each relaxosome protein as defined by Ilangovan *et al*., Frost *et al*. and Lum *et al*.[6-8] for IHF (*IHFa* and *IHFb*), TraM (*sbmA-C*), TraY (*sbyA* and *sbyC*), and TraI ($traI_{helicase}$ and $traI_{TE}$) are indicated under $oriT_{316}$ using boxes colour-coded red for IHF, yellow for TraY, dark blue, and orange for $\mathrm{TraI_{helicase}}$ and $\mathrm{TraI_{TE}}$, magenta for TraM, and black for the *nic* site.

c, Domain structure of TraI (top and middle) and the two binding modes of TraI. Top: TraI domains mapped on primary sequence. TE, VH, AH, and CTD are shown in orange, green, violet, and grey, respectively. Boundary residue numbers are shown. Middle: sub-domains of VH mapped on primary sequence. Boundary residues for NTD, 1A, 2A, and 2B/2B-like are indicated. Bottom: TraI binds sequence of the T-strand 3' to *nic* in its compact, protease-resistant, helicase mode (termed "$\mathrm{TraI_{helicase}}$"), while it binds sequences of the T-strand 5' to *nic* in its TE mode ("$\mathrm{TraI_{TE}}$").

d, Structure of $\mathrm{TraI_{helicase}}$ (PDB ID 5N8O). This structure was obtained using a 22-mer ssDNA (in magenta). Upper left and right: two views of TraI, one in ribbon and one in surface representations. Various domains and the DNA are labelled. Linker between VH and AH is shown in grey. The CTD was not part of this structure. Bottom: structure of the VH domain showing the location of the various subdomains. Color-coding is as in panel c.

e, Structure of *Escherichia coli* IHF bound to an IHF-binding site on phage lambda DNA (PDB ID 1IHF). IHF binding generates a bend of > 160° on dsDNA.

f, Composite model of pED208 TraM bound to *sbmA* (PDB ID 3ON0) and F $\mathrm{TraD_{711\text{-}717}}$ (PDB ID 3D8A). Protein and DNA are shown in ribbon, and $\mathrm{TraD_{711\text{-}717}}$ residues in red sticks. Each chain in the TraM tetramer is shown in a different colour.

Supplementary Fig. 2

ds_{-27_+143}

R-strand 5'TTGGTGGTTCTCACCACCAAAAGCACCACACCCCACGC**AAAAACAAGTTTTTGCTGATTTTTCTTTATAAATAGAGTGTTATGAAAAATTAGTTTCT**

-20 -10 -1 +1 +10 +20 +30 +40 +50 +60 +70

T-strand 3'AACCACCAAGAGTGGTGGTTTTCGTGGTGTGGGGTGCG**TTTTTGTTCAAAAACGACTAAAAAGAAATATTTATCTCACAATACTTTTTAATCAAAGA**

R-strand **CTTACTCTCTTTATGATATTTAAA**AAAGCGGTGTCGGCGCGGCTACAACAACGCGCCGACACCGTTTTGTAGG 3'

+80 +90 +100 +110 +120 +130 +140

T-strand **GAATGAGAGAAATACTATAAATTT**TTTCGCCACAGCCGCGCCGATGTTGTTGCGCGGCTGTGGCAAAACATCC 5'

$ss_{-27\_+8}ds_{+9\_+143}$

R-strand 5' C**GCAAAAACAAGTTTTTGCTGATTTTTCTTTATAAATAGAGTGTTATGAAAAATTAGTTTCT**

-20 -10 -1 +1 +10 +20 +30 +40 +50 +60 +70

T-strand 3'AACCACCAAGAGTGGTGGTTTTCGT**GGTGTGGGGTGCGTTTTTGTTCAAAAACGACTAAAAAGAAATATTTATCTCACAATACTTTTTAATCAAAGA**

R-strand **CTTACTCTCTTTATGATATTTAA**AAAAGCGGTGTCGGCGCGGCTACAACAACGCGCCGACACCGTTTTGTAGG 3'

+80 +90 +100 +110 +120 +130 +140

T-strand **GAATGAGAGAAATACTATAAATT**TTTTCGCCACAGCCGCGCCGATGTTGTTGCGCGGCTGTGGCAAAACATCC 5'

$ss_{-27\_+3}ds_{+4\_+143}$

R-strand 5' CCCCAC**GCAAAAACAAGTTTTTGCTGATTTTTCTTTATAAATAGAGTGTTATGAAAAATTAGTTTCT**

-20 -10 -1 +1 +10 +20 +30 +40 +50 +60 +70

T-strand 3'AACCACCAAGAGTGGTGGTTTTCGT**GGTGTGGGGTGCGTTTTTGTTCAAAAACGACTAAAAAGAAATATTTATCTCACAATACTTTTTAATCAAAGA**

R-strand **CTTACTCTCTTTATGATATTTAAA**AAAGCGGTGTCGGCGCGGCTACAACAACGCGCCGACACCGTTTTGTAGG 3'

+80 +90 +100 +110 +120 +130 +140

T-strand **GAATGAGAGAAATACTATAAATTT**TTTCGCCACAGCCGCGCCGATGTTGTTGCGCGGCTGTGGCAAAACATCC 5'

$ss_{-27\_-3}ds_{-2\_+143}$

R-strand 5' CCACACCCCAC**GCAAAAACAAGTTTTTGCTGATTTTTCTTTATAAATAGAGTGTTATGAAAAATTAGTTTCT**

-20 -10 -1 +1 +10 +20 +30 +40 +50 +60 +70

T-strand 3'AACCACCAAGAGTGGTGGTTTTCGT**GGTGTGGGGTGCGTTTTTGTTCAAAAACGACTAAAAAGAAATATTTATCTCACAATACTTTTTAATCAAAGA**

R-strand **CTTACTCTCTTTATGATATTTAAA**AAAGCGGTGTCGGCGCGGCTACAACAACGCGCCGACACCGTTTTGTAGG 3'

+80 +90 +100 +110 +120 +130 +140

T-strand **GAATGAGAGAAATACTATAAATTT**TTTCGCCACAGCCGCGCCGATGTTGTTGCGCGGCTGTGGCAAAACATCC 5'

$ss_{-27\_-8}ds_{-7\_+143}$

R-strand 5' AAGCACCACACCCCACGCAAAAACAAGTTTTTGCTGATTTTTCTTTATAAATAGAGTGTTATGAAAAATTAGTTTCT

-20 -10 -1 +1 +10 +20 +30 +40 +50 +60 +70

T-strand 3'AACCACCAAGAGTGGTGGTTTTCGTGGTGTGGGGTGCGTTTTTGTTCAAAAACGACTAAAAAGAAATATTTATCTCACAATACTTTTTAATCAAAGA

R-strand CTTACTCTCTTTATGATATTTAAAAAAGCGGTGTCGGCGCGGCTACAACAACGCGCCGACACCGTTTTGTAGG 3'

+80 +90 +100 +110 +120 +130 +140

T-strand GAATGAGAGAAATACTATAAATTTTTTCGCCACAGCCGCGCCGATGTTGTTGCGCGGCTGTGGCAAAACATCC 5'

$ss_{-27\_-13}ds_{-12\_+143}$

R-strand 5' ACCAAAAGCACCACACCCCACGCAAAAACAAGTTTTTGCTGATTTTTCTTTATAAATAGAGTGTTATGAAAAATTAGTTTCT

-20 -10 -1 +1 +10 +20 +30 +40 +50 +60 +70

T-strand 3'AACCACCAAGAGTGGTGGTTTTCGTGGTGTGGGGTGCGTTTTTGTTCAAAAACGACTAAAAAGAAATATTTATCTCACAATACTTTTTAATCAAAGA

R-strand CTTACTCTCTTTATGATATTTAAAAAAGCGGTGTCGGCGCGGCTACAACAACGCGCCGACACCGTTTTGTAGG 3'

+80 +90 +100 +110 +120 +130 +140

T-strand GAATGAGAGAAATACTATAAATTTTTTCGCCACAGCCGCGCCGATGTTGTTGCGCGGCTGTGGCAAAACATCC 5'

ds_{-2_+113}

R-strand 5' CCACACCCCACGCAAAAACAAGTTTTTGCTGATTTTTCTTTATAAATAGAGTGTTATGAAAAATTAGTTTCT

-1 +1 +10 +20 +30 +40 +50 +60 +70

T-strand 3' GGTGTGGGGTGCGTTTTTGTTCAAAAACGACTAAAAAGAAATATTTATCTCACAATACTTTTTAATCAAAGA

R-strand CTTACTCTCTTTATGATATTTAAAAAAGCGGTGTCGGCGCGGC 3'

+80 +90 +100 +110

T-strand GAATGAGAGAAATACTATAAATTTTTTCGCCACAGCCGCGCCG 5'

ds_{-67_+113} (poly-dT15$_{-17_-3}$)

R-strand 5' CGTAAATAATTTAACCCACTCCACAAAAAGGCTCAACAGG

-60 -50 -40 -30

T-strand 3' GCATTTATTAAATTGGGTGAGGTGTTTTTCCGAGTTGTCC

TTTTTTTTTTTTTTT

R-strand TTGGTGGTTC CCACACCCCACGCAAAAACAAGTTTTTGCTGATTTTTCTTTATAAATAGAGTGTTATGAAAAATTAGTTTCT

-20 -10 -1 +1 +10 +20 +30 +40 +50 +60 +70

T-strand AACCACCAAGAGTGGTGGTTTTCGTGGTGTGGGGTGCGTTTTTGTTCAAAAACGACTAAAAAGAAATATTTATCTCACAATACTTTTTAATCAAAGA

R-strand CTTACTCTCTTTATGATATTTAAAAAAGCGGTGTCGGCGCGGC 3'

+80 +90 +100 +110

T-strand GAATGAGAGAAATACTATAAATTTTTTCGCCACAGCCGCGCCG 5'

Supplementary Fig. 2. DNAs used in this study. *nic* is indicated by an arrow. The region of DNA for which a model could be derived from the density is shown in bold. Orange box locates the TE-binding site on the T-strand between bp_{-2} and bp_{+9}.

Supplementary Fig. 3

Data processing workflow for ds_{-27_+143}-R maps

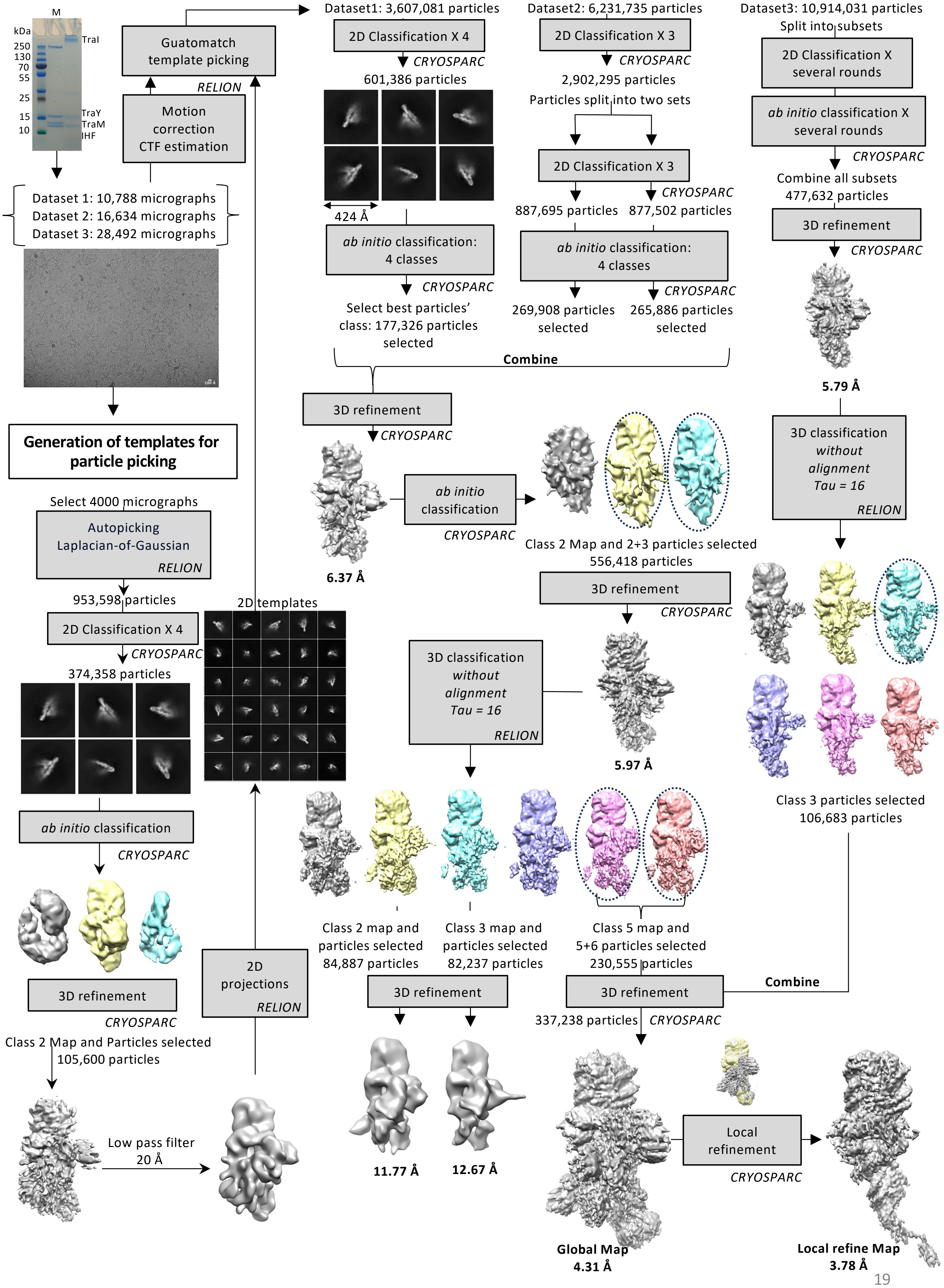

Supplementary Fig. 3. Image processing workflow for the cryo-EM structure determination of the ds$_{-27_+143}$-R complex. This being the first to be determined for any relaxosome structure, the *ab-initio* determination workflow is provided. For the uncropped gel image see source data file.

Supplementary Fig. 4

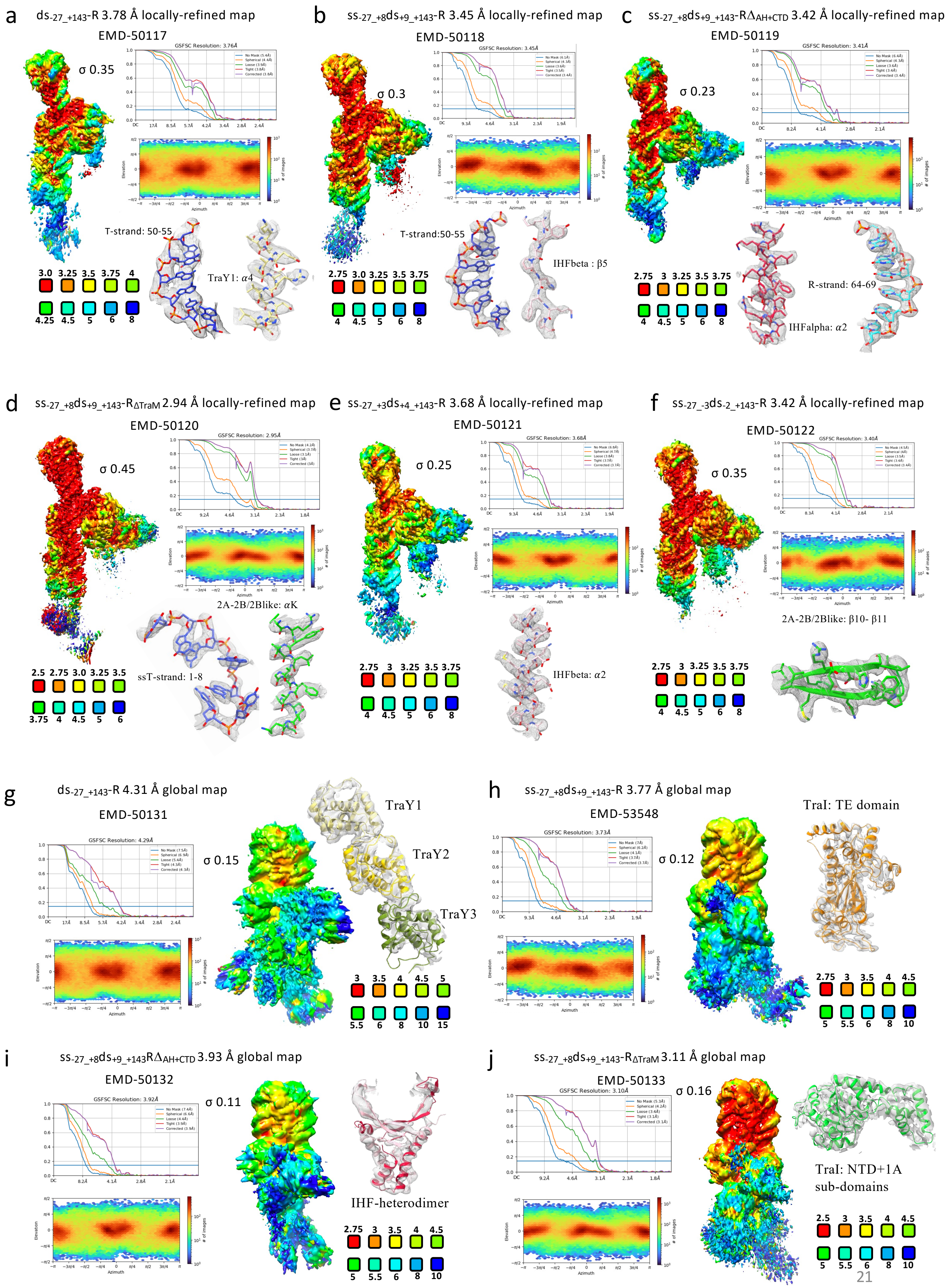

Supplementary Fig. 4. Cryo-EM maps used in this study. Maps are termed "locally-refined" or "global", depending on whether local refinement using a mask encompassing the DNA, the three TraYs, IHF, TraI $VH_{2A+2B/2B\text{-like}}$ and $TraI_{TE}$ has been applied or not, respectively. For each map, the density coloured by local resolution, the average resolution derived from Fourier Shell Correlation (FSC), the angular distribution and a representative region of the electron density map with the final model of the relaxosome built in it (in stick representation colour-coded as in Fig. 2e) are shown. Local resolution was calculated using CRYOSPARC (FSC cut-off 0.143) and coloured as indicated in the scale below the map. For each map, FSC plots show curves for correlation between 2 independently refined half-maps with no mask (blue), spherical mask (orange), loose mask (green), tight mask (red) and corrected (purple). Cut-off 0.143 (blue line) was used for resolution estimation.

a, ds_{-27_+143}-R locally-refined 3.78 Å map;

b, $ss_{-27\_+8}ds_{+9\_+143}$-R 3.45 Å locally-refined map;

c, $ss_{-27\_+8}ds_{+9\_+143}$-$R\Delta_{AH+CTD}$ 3.42 Å locally-refined map;

d, $ss_{-27\_+8}ds_{+9\_+143}$-$R\Delta_{TraM}$ 2.94 Å locally-refined map;

e, $ss_{-27\_+3}ds_{+4\_+143}$-R 3.68 Å locally-refined map;

f, $ss_{-27\_-3}ds_{-2\_+143}$-R 3.42 Å locally-refined map;

g, ds_{-27_+143}-R global 4.31 Å map;

h, $ss_{-27\_+8}ds_{+9\_+143}$-R 3.77 Å global map;

i, $ss_{-27\_+8}ds_{+9\_+143}$-$R\Delta_{AH+CTD}$ 3.93 Å global map;

j, $ss_{-27\_+8}ds_{+9\_+143}$-$R\Delta_{TraM}$ 3.11 Å global map.

Supplementary Fig. 5

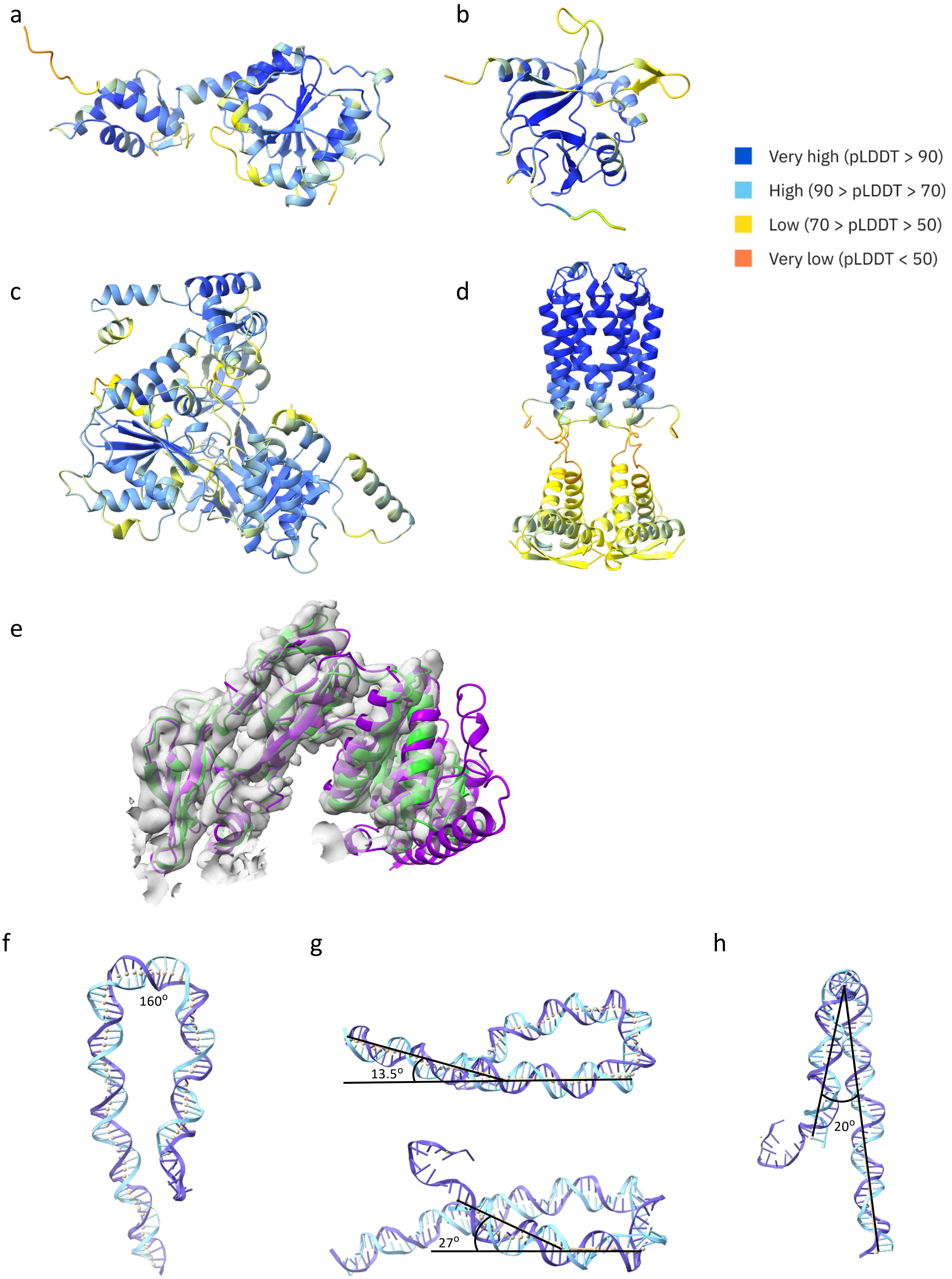

Supplementary Fig. 5. AlphaFold2 model used in this study and assessment of dsDNA bending from the $ss_{-27\_+8}ds_{+9\_+143}$-R structure.

For a-d, AlphaFold2 prediction models used to generate the $ss_{-27\_+8}ds_{+9\_+143}$-R global relaxosome, coloured by model confidence metric (pLDDT) on a scale from 0 to 100, where colour indicates the level of the pLDDT score.

a, TraI VH_{NTD+1A}; residues 307-563.

b, TraI CTD; residues 1473-1630.

c, TraI AH; residues 835-1459.

d, TraM tetramer.

e, Models of $VH_{2A+2B/2Blike}$ and $AH_{2A+2B/2Blike}$ sub-domains shown as cartoon representation and in green and violet, respectively. These are shown fitted in the density of ds_{-27_+143}-R locally refined map to confirm the accuracy of building $VH_{2A+2B/2Blike}$ in this density.

f, Bending induced by IHF binding evaluated as in Rice *et al.*[9].

g, Bending within the two DNA hairpin arms. Top panel: Bending triggered by the binding of the TraY train on the TraY arm of the DNA hairpin. Bottom panel: Bending in the *nic* arm is the result of sequence-induced bending between base pairs +20 and +34[10] and binding of $VH_{2A+2B/2B\text{-}like}$. Angles are reported.

h, Bending between the two DNA hairpin arms. When the DNA is viewed such that the IHF bend is perpendicular to the plane of view, the arms of the hairpin make an angle of 20°, seemingly moving away from each other.

Supplementary Fig. 6

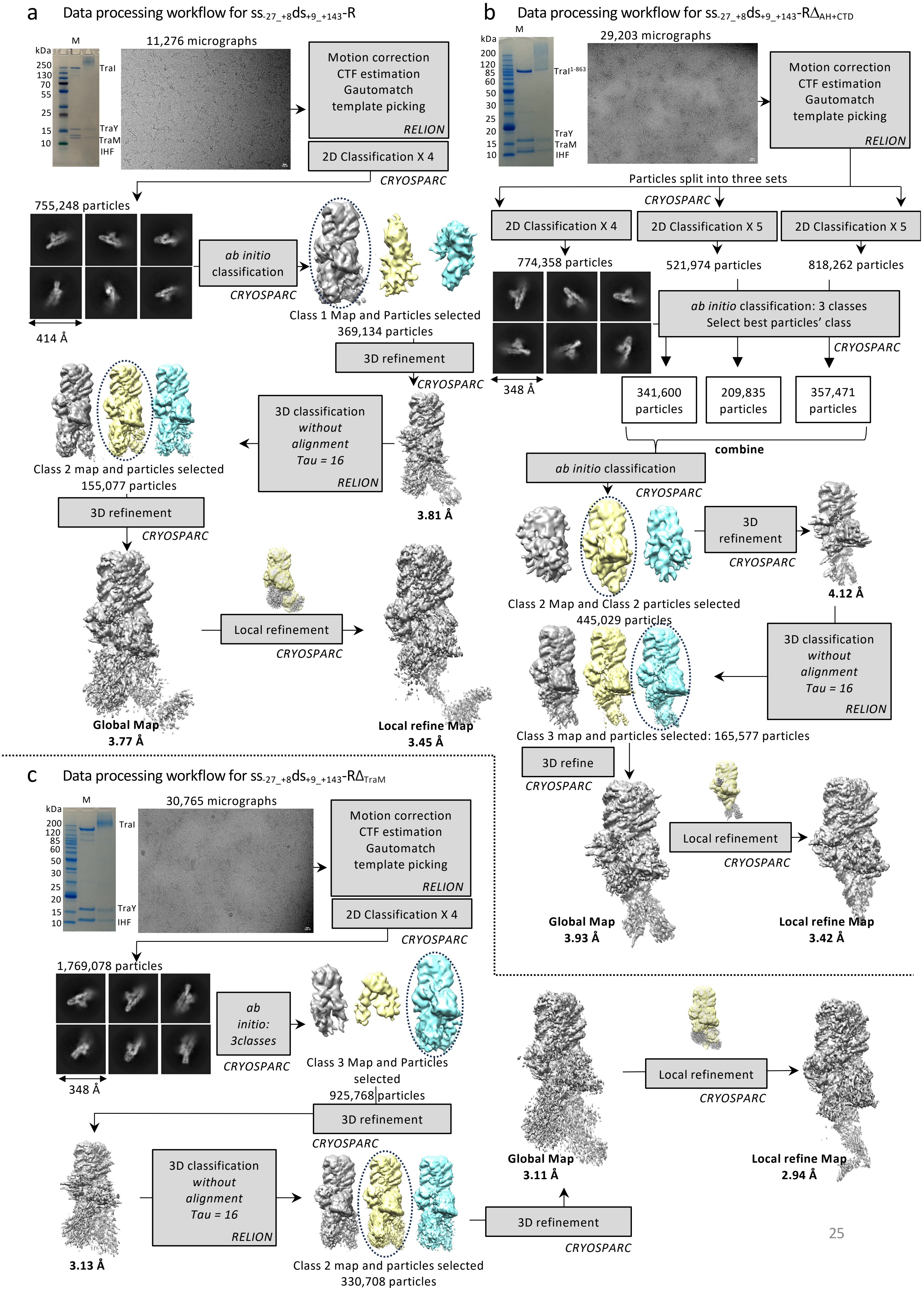

Supplementary Fig. 6. Image processing workflow for the cryo-EM structure determination of the (a) ss$_{-27_+8}$ds$_{+9_+143}$-R, (b) ss$_{-27_+8}$ds$_{+9_+143}$-RΔ_{AH+CTD} and (c) ss$_{-27_+8}$ds$_{+9_+143}$-RΔ_{TraM}. Workflow for the maps shown in supplementary Fig. 4e (ss$_{-27-+3}$ds$_{+4_+143}$-R 3.68 Å locally-refined map) and 4f (ss$_{-27_-3}$ds$_{-2_+143}$-R 3.42 Å locally-refined map) are not provided as they are essentially similar to that provided for ss$_{-27_+8}$ds$_{+9_+143}$-R. For the uncropped gel images see source data file.

Supplementary Fig. 7

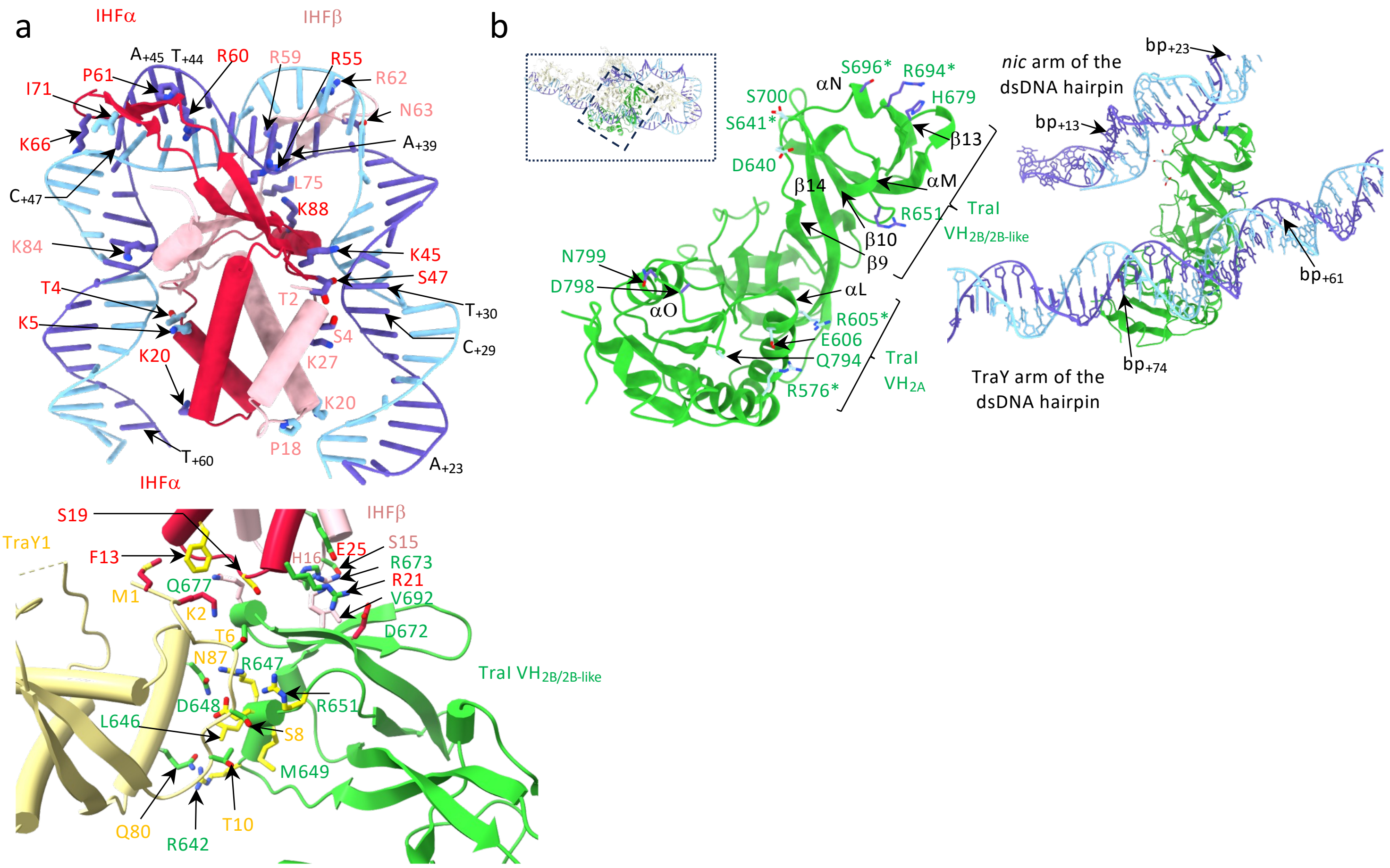

Supplementary Fig. 7. Details of Hub1 of the fully-assembled ss$_{-27_+8}$ds$_{+9_+143}$-R structure.

a, Details of interactions in Hub1. Representation, colour-coding, and labelling is as in Fig. 4b, except for interacting residues which are shown in stick representation and colour-coding according to the colour of the DNA or protein they interact with.

b, Details of interactions between TraI VH$_{2A+2B/2B\text{-like}}$ and DNA. Left: TraI VH$_{2A+2B/2B\text{-like}}$ residues involved in dsDNA-binding are shown in stick representation colour-coded in light and dark blue when interacting with the R- or T-strand, respectively. Right: same view as at left, with DNA and binding-site boundaries shown. The two arms of the dsDNA hairpin are indicated.

Supplementary Fig. 8

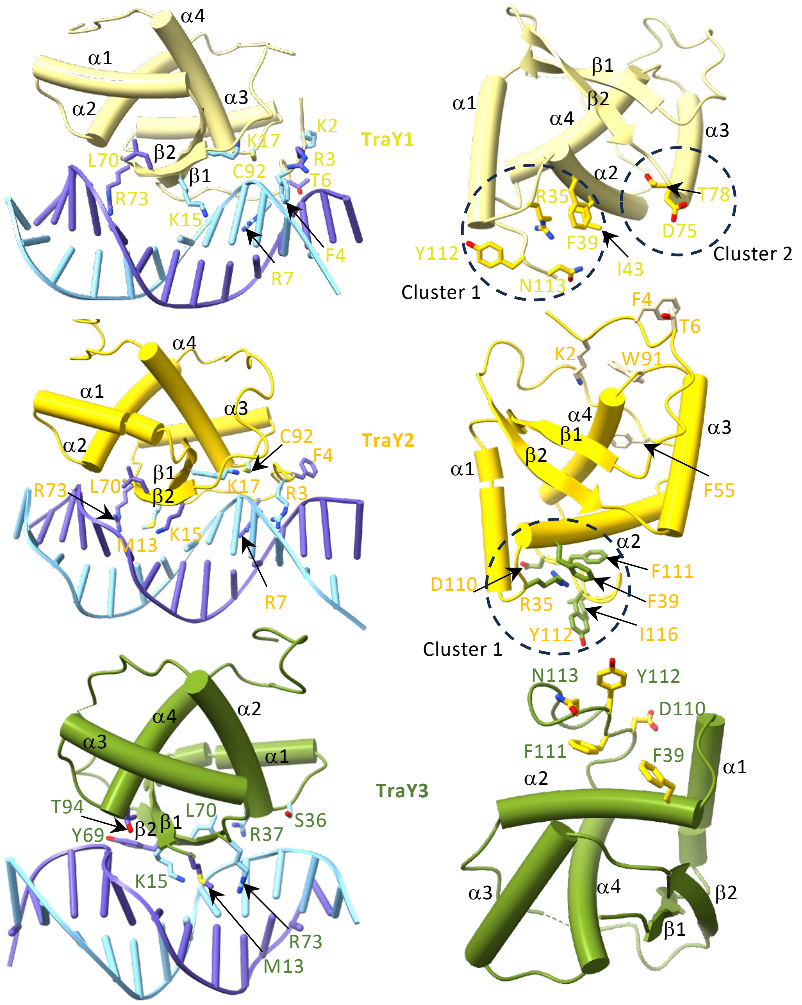

Supplementary Fig. 8. Details of the Hub2 of the fully-assembled $ss_{-27\_+8}ds_{+9\_+143}$-R structure.

Details of interactions in Hub2. Representation, colour-coding, and labelling is as in Fig. 5a, except for interacting residues which are shown in stick representation and colour-coded according to the colour of the DNA strand (left panel) or protein (right panel) they interact with. Left: TraY-DNA interactions for TraY1 (top), TraY2 (middle), TraY3 (bottom). Labelling of residues is as for the protein but their stick colour is that of interacting DNA strand, light blue for the R-strand and dark blue for the T-strand. Right: protein-protein interactions for TraY1 (top), TraY2 (middle), and TraY3 (bottom). Labelling of residues are as at left but stick colour is that of the protein the residue interacts with.

TraY's binding to DNA is typical of RHH dimers with the β1 and β2 strands interacting with the major groove of the DNA binding site[11]. But binding of TraY1 and TraY2 extends to the minor groove as well with residues at the extended N-terminus (before β1) contributing to minor groove binding. The third TraY (TraY3) binding site on DNA was predicted to be inverted and indeed TraY3 binds in an inverted orientation compared to TraY1 and TraY2. This agreed with a study by Lum *et al.*[8] which showed TraY binding to three sub-sites on *sbyA* (Fig. 1c and supplementary Fig. 1b). As a result, the N-terminal residues involved in minor groove binding in TraY1 and TraY2 are no longer involved. Also, residues in α1 and α2, which in TraY1 and TraY2 are located away from the DNA binding site, are now involved in DNA-binding.

TraY1 interaction with TraY2 involves two clusters of residues: the first at the C-terminus and in α1, α2, and α1-α2 (circle labelled "cluster 1") and another in β2-α3 and α3 (circle labelled "cluster 2"). Both regions contact TraY2 via the N-terminus of TraY2 but also its α2-β2 loop and α3. However, TraY2 interacts completely differently with TraY3 because TraY3 is inverted due to binding to the inverted DNA repeat. In fact, the TraY2-TraY3 interaction involves the same parts of the two proteins, namely cluster 1 mentioned above. Thus, cluster 1 of TraY2 and TraY3 face head to head in a manner that is conducive to complementary interactions between hydrophobic residues (F111, F39 for example) and charged residues to make ion pairs (D110, R35 for example).

Supplementary Fig. 9

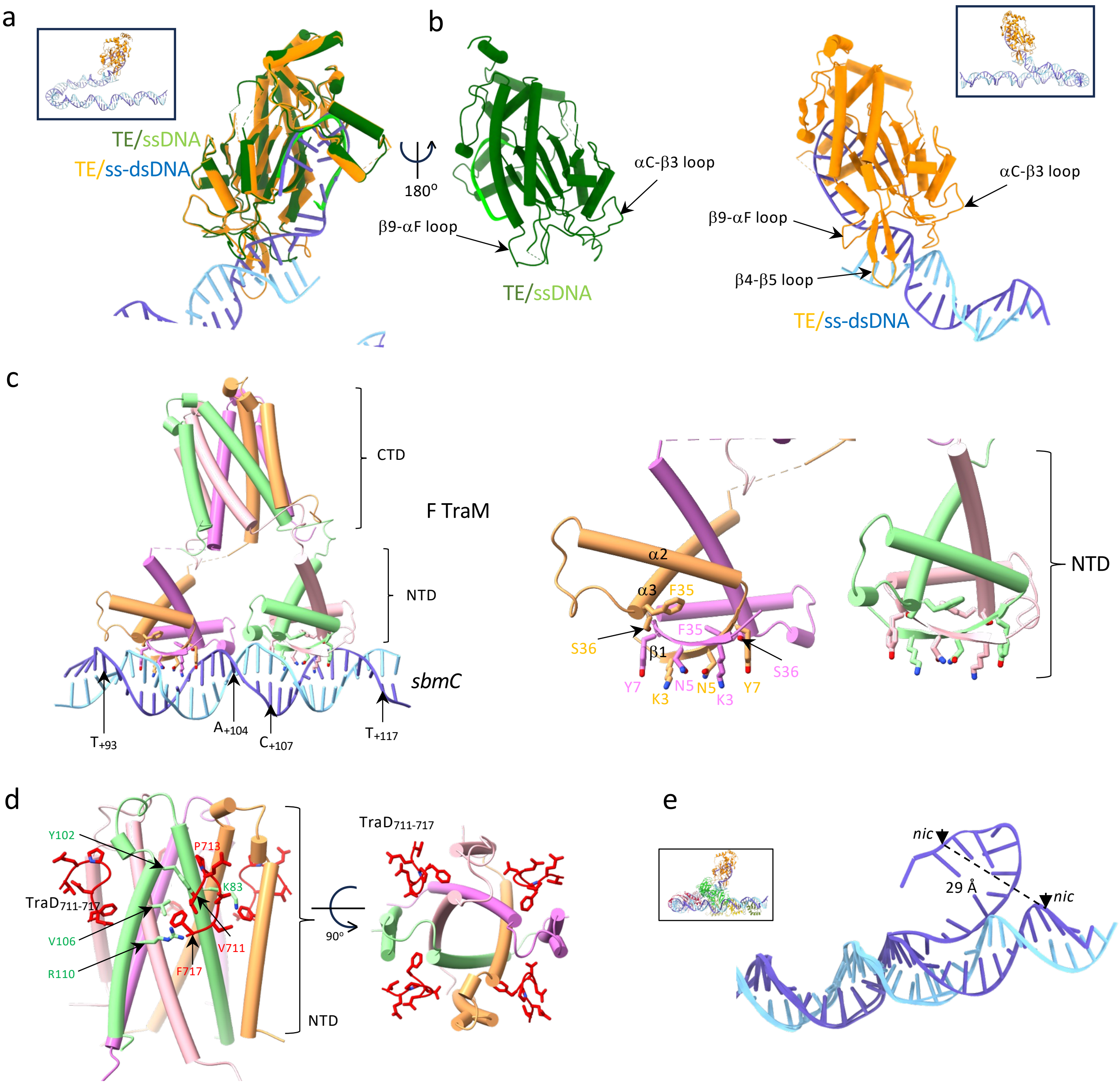

Supplementary Fig. 9. Interactions of TraI, TraM with TraD and *oriT* conformational change.

a, Superposition and comparison of TraI TE bound to $ss_{-27\_+8}ds_{+9\_+143}$ DNA (TE/ss-dsDNA) and the previous crystal structure of TraI TE bound to ss_{+9_-2} determined previously (TE/ssDNA; PDB ID 2A0I). RMSD in Cα position of 0.649 Å.

b, Secondary structures being re-organised or ordered upon binding of the ds region of $ss_{-27\_+8}ds_{+9\_+143}$ in the $ss_{-27\_+8}ds_{+9\_+143}$-R relaxosome structure. Left: TE/ssDNA structure. Right: TE/ss-dsDNA structure. The part of the structure undergoing conformational changes are labelled.

c, TraM binding to *sbmC*. Protein and DNA are shown in ribbon, colour-coded in four different colours for the 4 different TraM chains. R- and T-strand are in light and dark blue ribbon, respectively. Left: overall view of TraM binding. Boundary base pairs for the two TraM NTD dimer binding sites are indicated. Right: Residues involved in binding. Only residues for the binding site at left are labelled.

d, $TraD_{711-717}$ binding to TraM. The co-crystal structure of TraM-TraD (PDB ID: 3D8A) was used with the same colour-coding for TraM as above. TraM is shown in ribbon except for the residues involved in binding which are shown in stick. $TraD_{711-717}$ is shown in stick colour-coded red. Residues involved in interactions are labelled.

e, The $ss_{-27\_+8}ds_{+9\_+143}$ DNA in the $ss_{-27\_+8}ds_{+9\_+143}$-R structure superposed on the ds_{-27_+143} DNA in ds_{-27_+143} -R structure where the ds_{-27_+143} DNA has been extended to include the *nic* site. This superposition highlights the very large motion undergone by *nic* and its DNA region upon binding TraI TE.

Supplementary Fig. 10

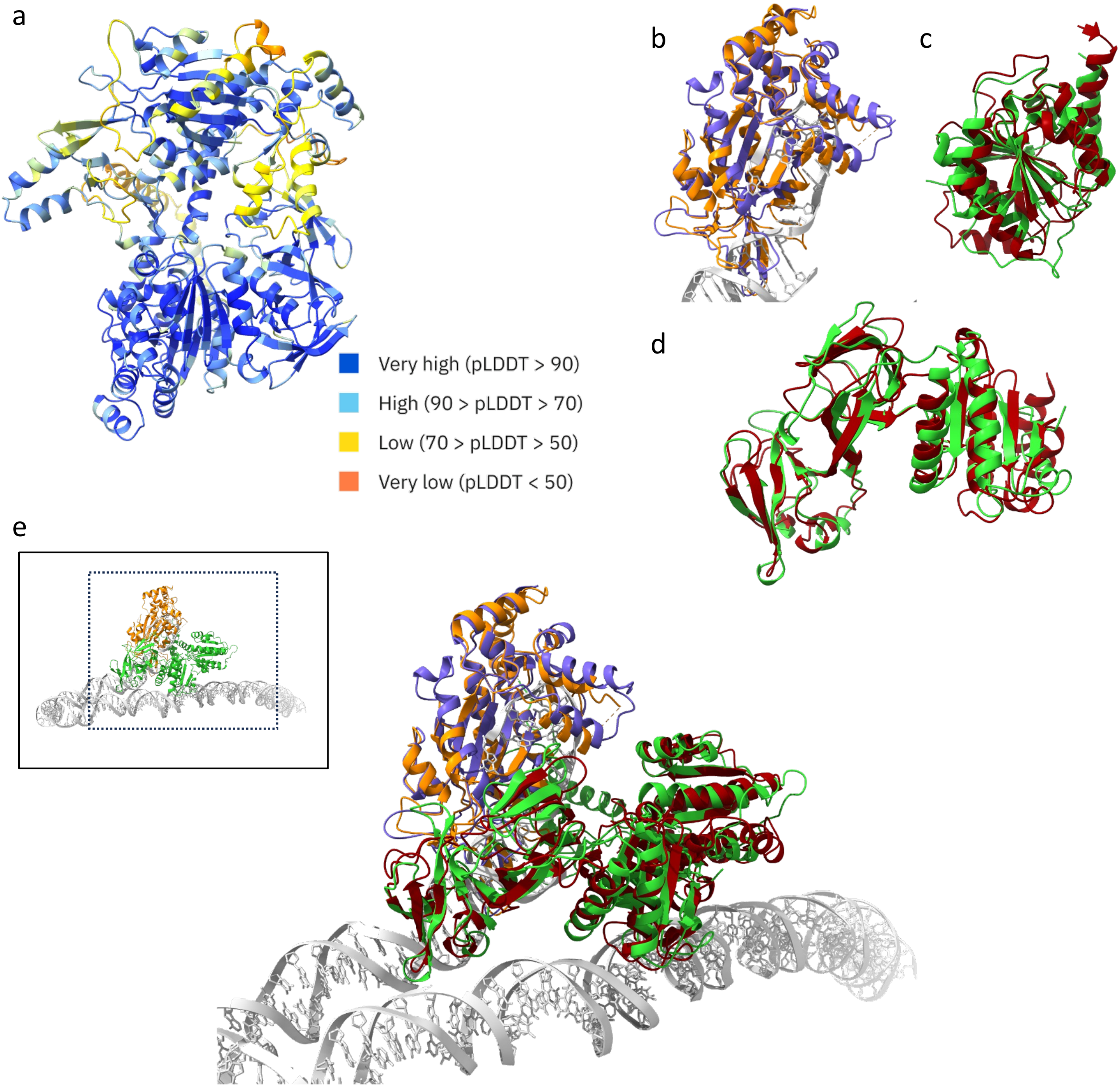

Supplementary Fig. 10. Proposed model for R388 plasmid TrwC

In b-f, color code of TraI domain is as in main text. Color code for TrwC TE and AH domains is in slate blue and red, respectively.

a, AlphaFold2 prediction model of relaxase TrwC of R388 plasmid in its $\mathrm{TrwC_{helicase}}$ mode and in the closed conformation, coloured by model confidence metric (pLDDT) on a scale from 0 to 100, where colour indicates the level of the pLDDT score.

b, Superpositions between TraI TE and TrwC TE (Cα RMSD of 2.6Å).

c, Superpositions between TraI $\mathrm{VH_{1A}}$ and TrwC $\mathrm{AH_{1A}}$ (Cα RMSD of 4.7 Å).

d, Superpositions between TraI $\mathrm{VH_{2A+2B/2B\text{-}like}}$ and TrwC $\mathrm{AH_{2A+2B/2B\text{-}like}}$ (Cα RMSD of 3.9 Å).

e, Superposition of $\mathrm{TrwC_{TE}}$ mode (open conformation) on $\mathrm{TraI_{TE}}$ mode. Insets of the entire $\mathrm{ss_{-27\_+8}ds_{+9\_+143}}$-R structure are provided for orientation. Within inset, dash-lined box shows the region detailed in the corresponding panel. Regions of $\mathrm{ss_{-27\_+8}ds_{+9\_+143}}$-R not being compared are not shown for improved clarity.

Supplementary References

1 Woodcock, D. M. *et al.* Quantitative evaluation of *Escherichia coli* host strains for tolerance to cytosine methylation in plasmid and phage recombinants. *Nucleic Acids Res* **17**, 3469-3478 (1989). https://doi.org:10.1093/nar/17.9.3469

2 Chandler, M. & Galas, D. J. IS1-mediated tandem duplication of plasmid pBR322. Dependence on recA and on DNA polymerase I. *J Mol Biol* **165**, 183-190 (1983). https://doi.org:10.1016/s0022-2836(83)80249-6

3 Lang, S. *et al.* Molecular recognition determinants for type IV secretion of diverse families of conjugative relaxases. *Mol Microbiol* **78**, 1539-1555 (2010). https://doi.org:10.1111/j.1365-2958.2010.07423.x

4 Amann, E., Ochs, B. & Abel, K. J. Tightly regulated tac promoter vectors useful for the expression of unfused and fused proteins in *Escherichia coli*. *Gene* **69**, 301-315 (1988). https://doi.org:10.1016/0378-1119(88)90440-4

5 Haft, R. J. *et al.* General mutagenesis of F plasmid TraI reveals its role in conjugative regulation. *J Bacteriol* **188**, 6346-6353 (2006). https://doi.org:10.1128/JB.00462-06

6 Ilangovan, A. *et al.* Cryo-EM Structure of a Relaxase Reveals the Molecular Basis of DNA Unwinding during Bacterial Conjugation. *Cell* **169**, 708-721 e712 (2017). https://doi.org:10.1016/j.cell.2017.04.010

7 Frost, L. S., Ippen-Ihler, K. & Skurray, R. A. Analysis of the sequence and gene products of the transfer region of the F sex factor. *Microbiol Rev* **58**, 162-210 (1994). https://doi.org:10.1128/mr.58.2.162-210.1994

8 Lum, P. L., Rodgers, M. E. & Schildbach, J. F. TraY DNA recognition of its two F factor binding sites. *J Mol Biol* **321**, 563-578 (2002). https://doi.org:10.1016/s0022-2836(02)00680-0

9 Rice, P. A., Yang, S., Mizuuchi, K. & Nash, H. A. Crystal structure of an IHF-DNA complex: a protein-induced DNA U-turn. *Cell* **87**, 1295-1306 (1996). https://doi.org:10.1016/s0092-8674(00)81824-3

10 Tsai, M. M., Fu, Y. H. & Deonier, R. C. Intrinsic bends and integration host factor binding at F plasmid oriT. *J Bacteriol* **172**, 4603-4609 (1990). https://doi.org:10.1128/jb.172.8.4603-4609.1990

11 Schreiter, E. R. & Drennan, C. L. Ribbon-helix-helix transcription factors: variations on a theme. *Nat Rev Microbiol* **5**, 710-720 (2007). https://doi.org:10.1038/nrmicro1717
