# Supplementary material for: Cryo-EM Structure of the relaxosome, a complex essential for bacterial mating and the spread of antibiotic resistance genes
Source: Nat Commun. 2025 May 27;16:4906. doi: 10.1038/s41467-025-60116-6 (PMC12117103; doi:10.1038/s41467-025-60116-6)
Supplement: Supplementary file 2 — Description of Additional Supplementary Files [file 41467_2025_60116_MOESM2_ESM.pdf]

Description of Additional Supplementary Files

File name: Supplementary Data 1
Description: A model of the fully-assembled $ss_{-27\_+8}ds_{+9\_+143}$-R relaxosome color coded as in the text and an associated ChimeraX session can be found with the Supplementary Data 1.
