# Supplementary material for: Cryo-EM Structure of the relaxosome, a complex essential for bacterial mating and the spread of antibiotic resistance genes
Source: Nat Commun. 2025 May 27;16:4906. doi: 10.1038/s41467-025-60116-6 (PMC12117103; doi:10.1038/s41467-025-60116-6)
Supplement: Supplementary file 6 — Source Data [file 41467_2025_60116_MOESM6_ESM.zip › SourceDataFiles/Uncropped Gel Images.pptx]

## Slide 1
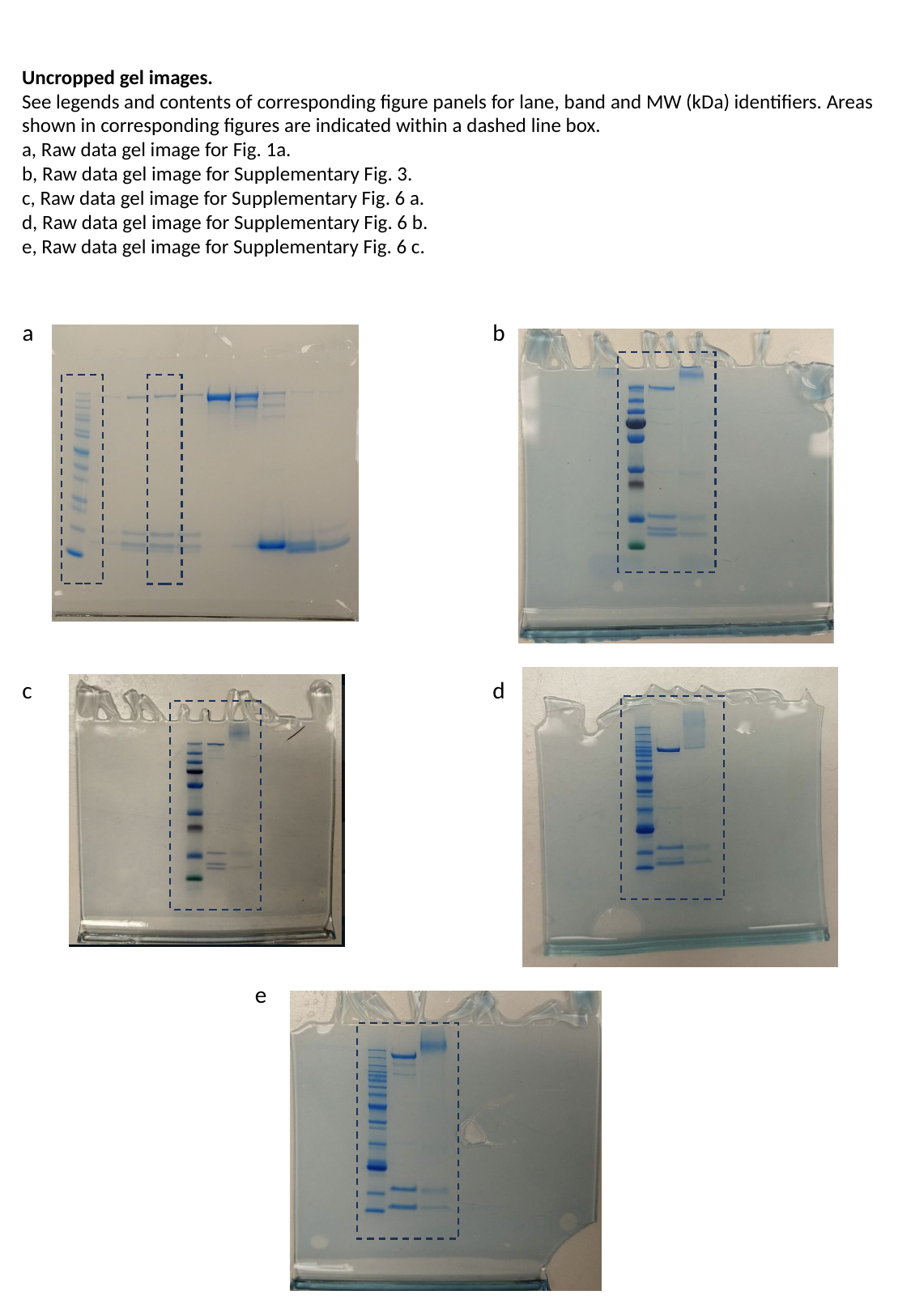

Uncropped gel images.
See legends and contents of corresponding figure panels for lane, band and MW (kDa) identifiers. Areas shown in corresponding figures are indicated within a dashed line box.
a, Raw data gel image for Fig. 1a.
b, Raw data gel image for Supplementary Fig. 3.
c, Raw data gel image for Supplementary Fig. 6 a.
d, Raw data gel image for Supplementary Fig. 6 b.
e, Raw data gel image for Supplementary Fig. 6 c.
a
b
c
d
e
